# Synthesis and structure of *trans*-2,5-di­methyl­piperazine-1,4-diium di­hydrogen diphosphate

**DOI:** 10.1107/S2056989024010132

**Published:** 2024-10-24

**Authors:** Houda Mrad, Adel Elboulali, Benoît Baptiste, Samah Akriche

**Affiliations:** a Laboratory of Materials Chemistry (LR13ES08), Faculty of Sciences of Bizerte, University of Carthage, 7021 Zarzouna, Bizerte, Tunisia; b Institut de Minéralogie, de Physique des Matériaux et de, Cosmochimie CNRS - UMR 7590, Plateforme de diffraction des rayons X, 75005 Paris, France; University of Aberdeen, United Kingdom

**Keywords:** crystal structure, Hirshfeld surface analysis, di­hydrogen diphosphate, *trans-*2,5-di­methyl­piperazine

## Abstract

In the title salt, C_6_H_16_N_2_^2+^ ·H_2_P_2_O_7_^2−^, the complete dication is generated by a crystallographic centre of symmetry with the methyl groups in equatorial orientations. The complete dianion is generated by a crystallographic twofold axis with the central O atom lying on the axis: the P—O—P bond angle is 135.50 (12)°. In the crystal, the di­hydrogen diphosphate anions are linked by O—H⋯O hydrogen bonds, generating (001) layers. The organic cations bond to the inorganic layers by way of N—H⋯O and C—H⋯O hydrogen bonds. A Hirshfeld surface analysis shows that the most important contributions for the crystal packing are from O⋯H/H⋯O (60.5%) and H⋯H (39.4%) contacts.

## Abstract 2

In the title salt, the complete cation and anion are generated by crystallographic inversion and twofold symmetry, respectively. In the crystal, the di­hydrogen diphosphate anions are linked by O—H⋯O hydrogen bonds, generating (001) layers. The organic cations bond to the inorganic layers by way of N—H⋯O and C—H⋯O hydrogen bonds.

## 1. Chemical context

Hybrid organic–diphosphate-based materials have received attention due to their role in catalytic, adsorption, ion-exchange, optical and biological processes (Chen & Munson, 2002 ▸; Ballarini *et al.*, 2006 ▸). Protonated diphosphate forms such as HP_2_O_7_^3–^ or H_2_P_2_O_7_^2–^ have the ability to associate with organic cations by means of ionic and non-covalent inter­actions (Desiraju, 1989 ▸; Steiner, 2002 ▸) to generate different supra­molecular architectures. Among the many diphosphate structures templated with organic cations are (C_2_H_10_N_2_)·H_2_P_2_O_7_ (Averbuch-Pouchot & Durif, 1993 ▸), (C_5_H_6_N_2_O_2_)_2_·H_2_P_2_O_7_ (Toumi Akriche *et al.*, 2010 ▸), (C_8_H_12_N)_2_·H_2_P_2_O_7_, (Marouani *et al.*, 2010 ▸ (C_8_H_12_NO)_2_·H_2_P_2_O_7_ (Elboulali *et al.*, 2013 ▸) and (C_6_H_5_CH_2_NH_3_)_2_·H_2_P_2_O_7_ (Saad *et al.*, 2014 ▸). As part of our ongoing studies of these compounds, we now report the synthesis and structure of the title compound, C_6_H_16_N_2_^2+^·H_2_P_2_O_7_^2–^, (I).
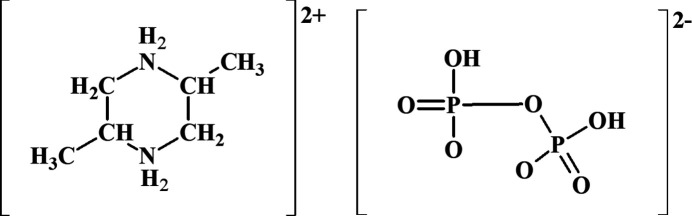


## 2. Structural commentary

**Figure 1:**
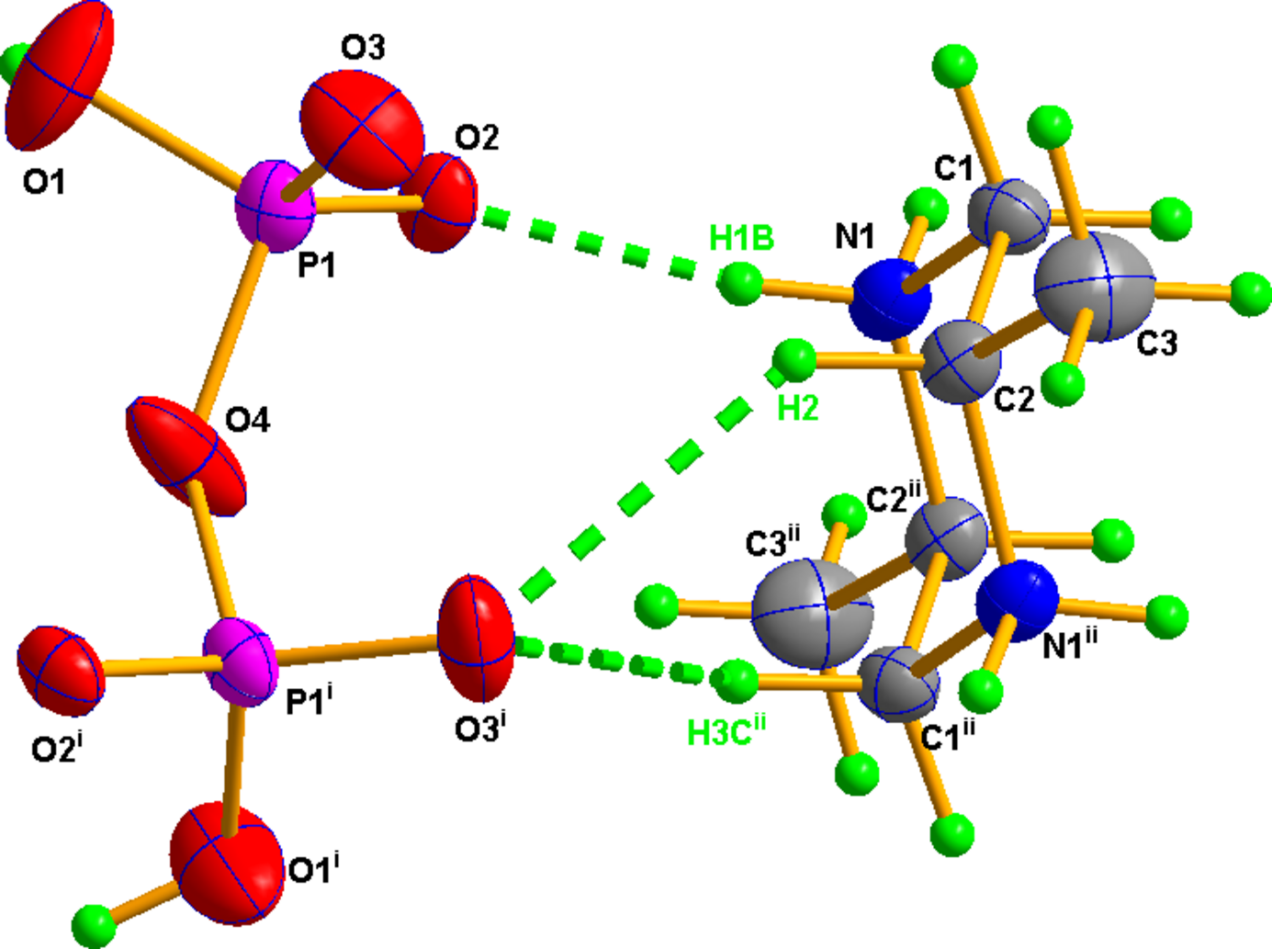
The mol­ecular structure of (I) with displacement ellipsoids drawn at the 50% probability level. Symmetry codes: (i) −*x* + 1, *y*, −*z* + 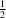; (ii) −*x* + 1, −*y* + 1, −*z* + 1. The H atoms are presented as small spheres of arbitrary radius. Hydrogen bonds are shown as green dotted lines.

The asymmetric unit of (I) comprises a half diphosphate anion completed by a crystallographic twofold rotation axis (atom O4 lies on the axis) and half a *trans-*2,5-di­methyl­piperazine-1,4-dium cation completed by inversion symmetry (Fig. 1 ▸). The phospho­rous atom adopts a distorted tetra­hedral geometry with bond lengths and angles in the range 1.4916 (11)–1.5884 (8) Å and 102.44 (9)–116.60 (8)°, respectively. The longest P—O distance corresponds to the bridging oxygen atom [P1—O4 = 1.5884 (8) Å], the inter­mediate one is the P—OH bond [P1—O1 = 1.5389 (15) Å], whereas the shortest bonds correspond to terminal oxygen atoms [P1—O2 = 1.4916 (11) and P1—O3 = 1.4924 (14) Å] in agreement with previous di­hydrogen diphosphate structures elaborated by our group (Toumi Akriche *et al.*, 2010 ▸; Saad *et al.*, 2014 ▸). The PO_4_ tetra­hedra are fused by atom O4 forming a bent P_2_O_7_ unit [P1—O4—P1^i^ = 135.50 (12)°; symmetry code: (i) −*x* + 1, *y*, −*z* + 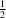)] and staggered conformation [O1—P1—P1^i^—O2^i^ = 42.8 (1)°], close to those previously observed for diphos­phates with twofold symmetry (Marouani *et al.*, 2010 ▸; Charfi & Jouini, 1997 ▸). In the cation, the piperazine ring adopts a chair conformation with puckering parameters *Q* = 0.2770 Å, θ = 90° and φ = 142° in which the methyl substituents occupy equatorial sites. The bonds and angles in the cation [N/C—C in the range 1.484 (2)–1.514 (2) Å and N/C—C/N—C, in the range 108.68 (11)–112.80 (12)°] show no significant difference from those reported in other *trans*-2,5-di­methyl­piperazine based salts (Landolsi & Abid, 2021 ▸; Gatfaoui *et al.*, 2014 ▸).

## 3. Supra­molecular features

**Figure 2:**
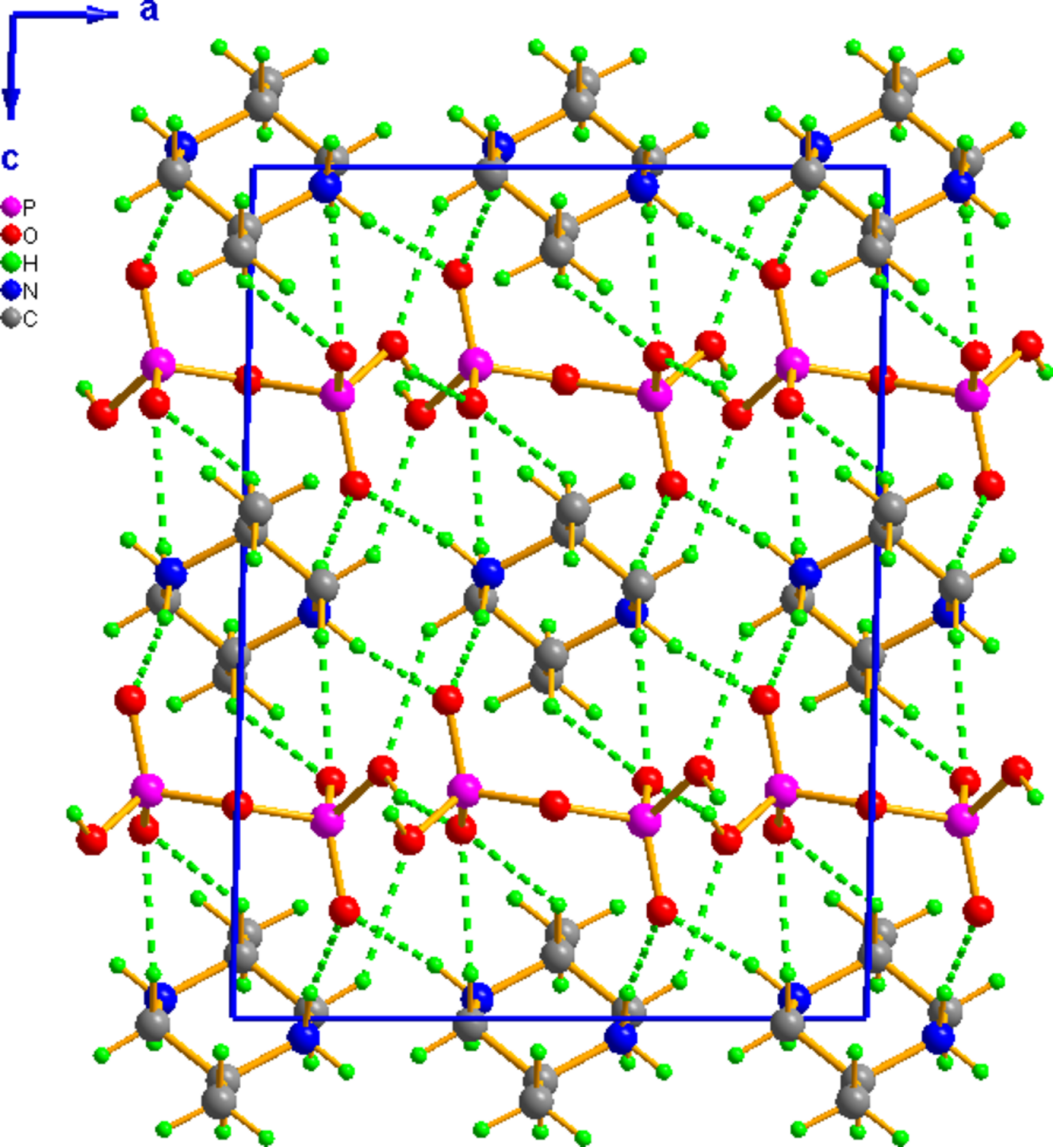
The projection along the *b*-axis direction of the crystal packing of (I). Hydrogen bonds are shown as dotted lines.

**Figure 3:**
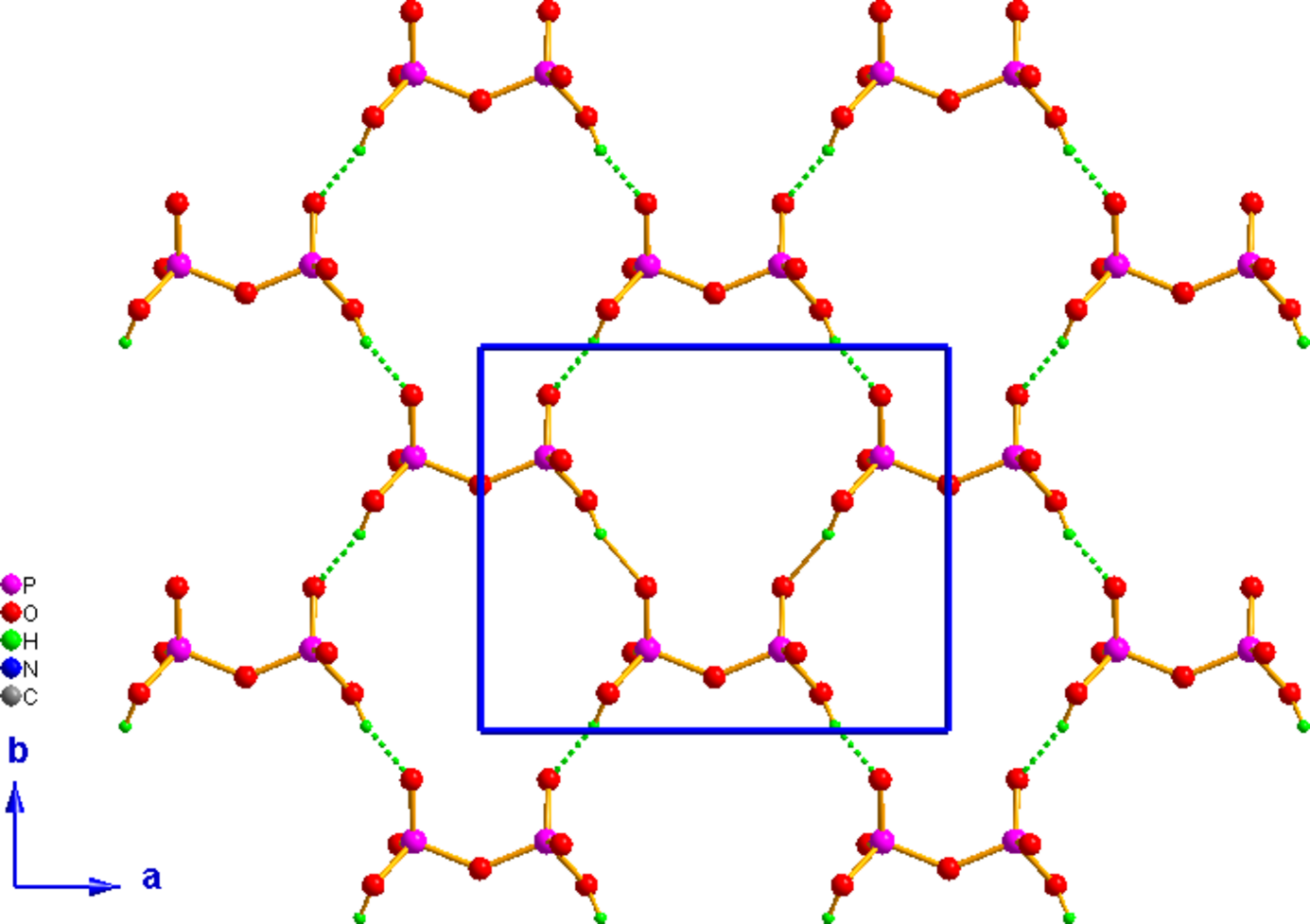
The layered di­hydrogen diphosphate self-assembly, viewed along the *c* axis at *z* = 1/4. The O—H⋯O hydrogen bonds are shown as green dotted lines.

The extended structure of (I) is built up from hydrogen-bonded sheets of diphosphate anions extended along the crystallographic *c*-axis direction at *z* = 1/4 and 3/4 with the cations lying between these anionic sheets featuring extensive hydrogen bonds, so as to build a three-dimensional supra­molecular network (Fig. 2 ▸). A projection along the *c* axis at *z* = 1/4 of the inorganic sheet (Fig. 3 ▸) shows that O—H⋯O hydrogen bonds (Table 1 ▸) link adjacent di­hydrogen diphos­phate anions to develop anionic layers extending parallel to (001). Electrostatic, medium-strength N—H⋯O and weaker C—H⋯O inter­actions (Table 1 ▸) between the organic cations and these anionic layers are responsible for the three-dimensional crystal structure.

## 4. Hirshfeld surface analysis

**Figure 4:**
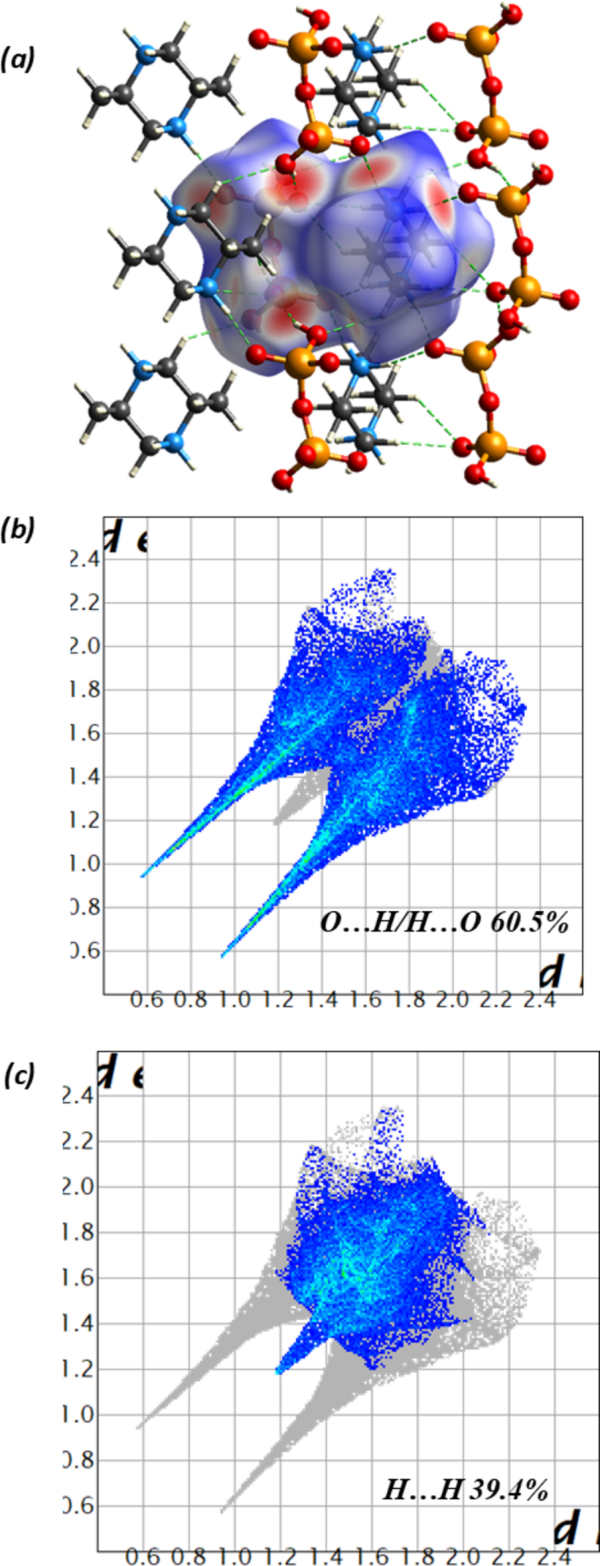
Hirshfeld surface (*a*) mapped with *d*_norm_ and fingerprint plots showing the major inter-contacts contributions of (*b*) O⋯H/H⋯O and (*c*) H⋯H.

Fig. 4 ▸*a* shows the Hirshfeld surface generated with Crystal Explorer (Wolff *et al.*, 2012 ▸) mapped over *d*_norm_ with red spots corresponding to short inter-atomic contacts. The fingerprint plots illustrated in Fig. 4 ▸*b* and 4*c* with characteristic spikes indicate that the major inter-atomic contributions to the structure are from O⋯H/H⋯O (60.5%) and H⋯H (39.4%) contacts in accordance with the structure topology.

## 5. Synthesis and experimental

The monocrystals of (I) were synthesized into two steps. Firstly, di­phospho­ric acid, H_4_P_2_O_7_, was obtained from Na_4_P_2_O_7_ (26 mg, 50 ml H_2_O) by using an ion-exchange resin (Amberlite IR 120). Then, the fresh di­phospho­ric acid solution was neutralized with *trans*-2,5-di­methyl­piperazine base in a 1:1 molar ratio at low temperature. The resulting solution was slowly evaporated at room temperature for several days until colourless needle-shaped crystals of (I) were grown.

## 6. IR spectrum

**Figure 5:**
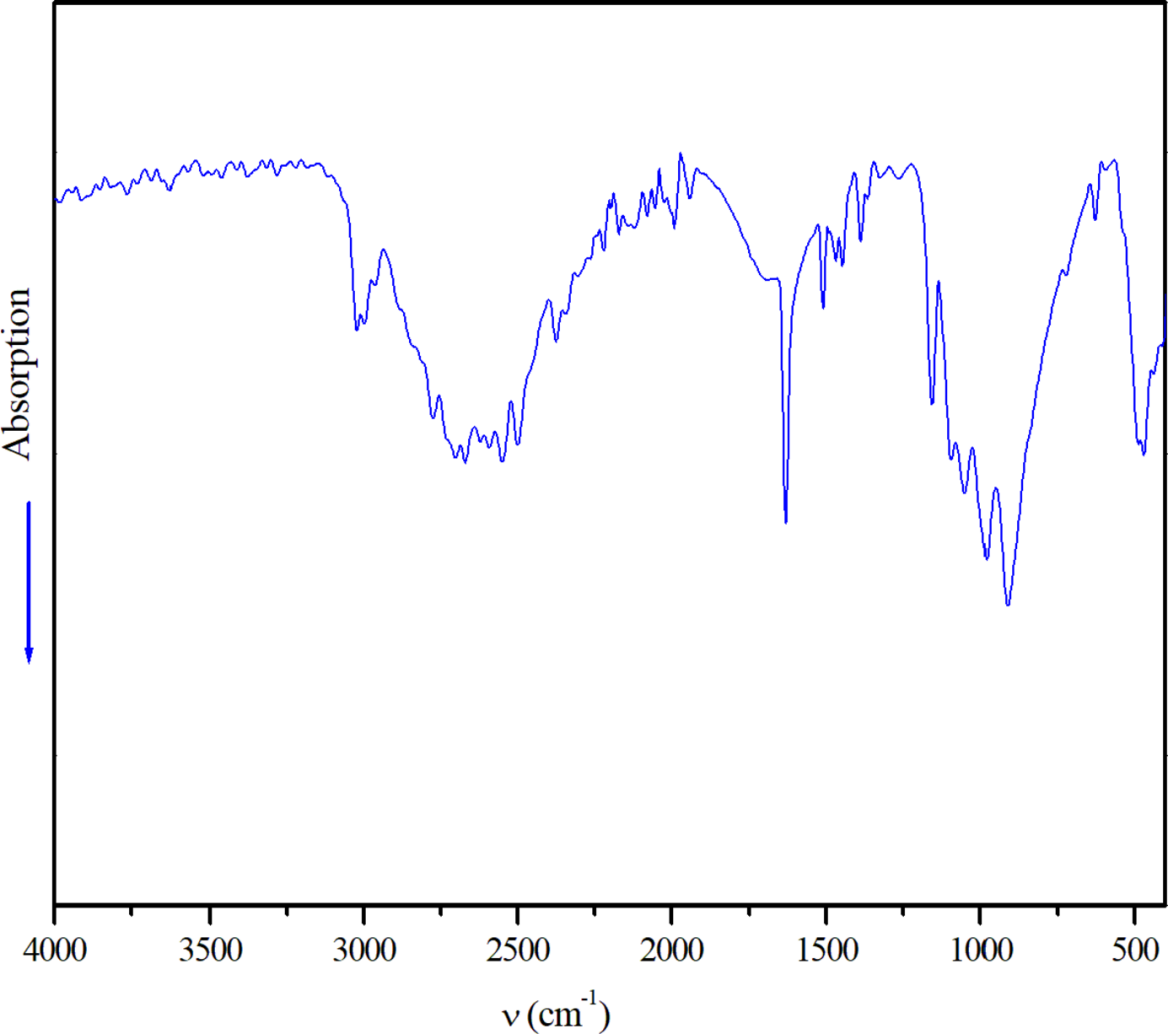
The infrared spectrum of (I).

The IR spectrum of (I) was collected at room temperature using a Perkin–Elmer Spectrum BXII spectrometer (KBr method) between 400 and 4000 cm^−1^ (Fig. 5 ▸). The spectrum exhibits broad bands between 2376 and 3027 cm^−1^, which can be assigned to the stretching modes of the –CH_3_, –CH_2_–, –CH– and (–NH_2_)^+^ groups of the organic cation (Silverstein *et al.*, 1974 ▸). The broadness of these bands in (I) is indicative of the presence of a hydrogen-bonding network. The bending vibrations of these groups are observed in the region 1321–1631 cm^−1^. The inter­nal modes of the (H_2_P_2_O_7_)^2–^ anion appear in the range 410–1265 cm^−1^ (Harcharras *et al.*, 1997 ▸; Kamoun *et al.*, 1992 ▸). The elongation modes of the PO_2_ and PO_3_ terminal groups occur between 1265 and 975 cm^−1^, whereas the terminal P—O stretching vibration of the PO_2_ group is observed at 1155 cm^−1^ and those at 1093 and 1051 cm^−1^ are attributed to the symmetric and asymmetric terminal P—O stretching vibration of the PO_3_ group (Sarr & Diop, 1987 ▸). The rocking of the PO_2_ and PO_3_ deformation modes occur between 491 and 627 cm^−1^. The symmetric and asymmetric elongation modes of the P—O—P bridge for the diphosphate group with a bent conformation are observed as ν_as_(P—O—P) = 911 and 975 cm^−1^ and ν_s_(P—O—P)= 721 cm^−1^. The simultaneous activity of these vibration modes confirms the results obtained by X-ray concerning the bent geometry and the *C*_2_ symmetry of the diphosphate anion. With regard to the Laza­rev (1972 ▸) correlation between the P—O—P bridge stretching frequencies and the bridge angle value as Δ = (ν_as_– ν_s_)/(ν_as_+ ν_s_) = 0.12, and by simple extrapolation of the graph of Δ = *f*(α) given by Rulmont *et al.* (1991 ▸), we can estimate the calculated P—O—P angle of 136° in excellent agreement with the value of 135.50 (12)° found for (I).

## 7. Refinement

Crystal data, data collection and structure refinement details are summarized in Table 2 ▸. All H atoms were located in a difference Fourier map and their positions were freely refined.

## Supplementary Material

Crystal structure: contains datablock(s) I. DOI: 10.1107/S2056989024010132/hb8111sup1.cif

Structure factors: contains datablock(s) I. DOI: 10.1107/S2056989024010132/hb8111Isup3.hkl

Supporting information file. DOI: 10.1107/S2056989024010132/hb8111Isup3.cml

CCDC reference: 2386600

Additional supporting information:  crystallographic information; 3D view; checkCIF report

## Figures and Tables

**Table 1:** Hydrogen-bond geometry (Å, °)

*D*—H⋯*A*	*D*—H	H⋯*A*	*D*⋯*A*	*D*—H⋯*A*
O1—H1⋯O3^i^	0.88 (3)	1.61 (3)	2.460 (2)	161 (3)
N1—H1*A*⋯O2^ii^	0.90 (2)	1.86 (2)	2.7454 (17)	167 (2)
N1—H1*B*⋯O2	0.90 (2)	1.87 (2)	2.7543 (17)	168 (2)
C1—H1*C*⋯O3^iii^	0.982 (19)	2.353 (19)	3.193 (2)	143.0 (15)
C1—H1*D*⋯O1^iv^	0.970 (19)	2.41 (2)	3.205 (2)	139.1 (15)
C2—H2⋯O3^v^	1.00 (2)	2.53 (2)	3.344 (2)	138.3 (15)

Symmetry codes: (i) 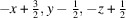; (ii) 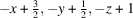; (iii) 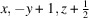; (iv) 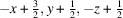; (v) 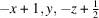.

**Table 2:** Experimental details

Crystal data	
Chemical formula	C_6_H_16_N_2_^2+^·H_2_O_7_P_2_^2−^
*M*_r_	292.16
Crystal system, space group	Monoclinic, *C*2/*c*
Temperature (K)	293
*a*, *b*, *c* (Å)	10.2557 (5), 8.3978 (5), 13.7681 (7)
β (°)	91.422 (4)
*V* (Å^3^)	1185.42 (11)
*Z*	4
Radiation type	Mo *K*α
μ (mm^−1^)	0.39
Crystal size (mm)	0.30 × 0.20 × 0.10

Data collection	
Diffractometer	Xcalibur diffractometer with Sapphire3 CCD detector
Absorption correction	Multi-scan (*CrysAlis PRO*; Rigaku OD, 2019 ▸)
*T*_min_, *T*_max_	0.979, 1.000
No. of measured, independent and observed [*I* > 2σ(*I*)] reflections	9376, 2255, 1738
*R*_int_	0.040
(sin θ/λ)_max_ (Å^−1^)	0.769

Refinement	
*R*[*F*^2^ > 2σ(*F*^2^)], *wR*(*F*^2^), *S*	0.041, 0.103, 1.05
No. of reflections	2255
No. of parameters	114
H-atom treatment	All H-atom parameters refined
Δρ_max_, Δρ_min_ (e Å^−3^)	0.59, −0.39

Computer programs: *CrysAlis PRO* (Rigaku OD, 2019 ▸), *SHELXS97* (Sheldrick, 2008 ▸), *SHELXL2018/3* (Sheldrick, 2015 ▸), *DIAMOND* (Brandenburg & Putz, 2005 ▸) and *WinGX* publication routines (Farrugia, 2012 ▸).

## supplementary crystallographic information

### trans-2,5-Dimethylpiperazine-1,4-diium dihydrogen diphosphate Crystal data

**Table:**

C_6_H_16_N_2_^2+^·H_2_O_7_P_2_^2^^−^	*F*(000) = 616
*M**_r_* = 292.16	*D*_x_ = 1.637 Mg m^−^^3^
Monoclinic, *C*2/*c*	Mo *K*α radiation, λ = 0.71073 Å
*a* = 10.2557 (5) Å	Cell parameters from 2302 reflections
*b* = 8.3978 (5) Å	θ = 4.0–31.8°
*c* = 13.7681 (7) Å	µ = 0.39 mm^−^^1^
β = 91.422 (4)°	*T* = 293 K
*V* = 1185.42 (11) Å^3^	Needle, colorless
*Z* = 4	0.30 × 0.20 × 0.10 mm

### trans-2,5-Dimethylpiperazine-1,4-diium dihydrogen diphosphate Data collection

**Table:**

Xcalibur diffractometer with Sapphire3 CCD detector	2255 independent reflections
Radiation source: fine-focus sealed X-ray tube, Enhance (Mo) X-ray Source	1738 reflections with *I* > 2σ(*I*)
Graphite monochromator	*R*_int_ = 0.040
Detector resolution: 16.0318 pixels mm^-1^	θ_max_ = 33.1°, θ_min_ = 3.5°
ω scans	*h* = −15→15
Absorption correction: multi-scan (CrysAlisPro; Rigaku OD, 2019)	*k* = −12→12
*T*_min_ = 0.979, *T*_max_ = 1.000	*l* = −21→21
9376 measured reflections	

### trans-2,5-Dimethylpiperazine-1,4-diium dihydrogen diphosphate Refinement

**Table:**

Refinement on *F*^2^	Primary atom site location: structure-invariant direct methods
Least-squares matrix: full	Secondary atom site location: difference Fourier map
*R*[*F*^2^ > 2σ(*F*^2^)] = 0.041	Hydrogen site location: inferred from neighbouring sites
*wR*(*F*^2^) = 0.103	All H-atom parameters refined
*S* = 1.05	*w* = 1/[σ^2^(*F*_o_^2^) + (0.038*P*)^2^ + 1.0885*P*] where *P* = (*F*_o_^2^ + 2*F*_c_^2^)/3
2255 reflections	(Δ/σ)_max_ = 0.001
114 parameters	Δρ_max_ = 0.59 e Å^−^^3^
0 restraints	Δρ_min_ = −0.38 e Å^−^^3^

### trans-2,5-Dimethylpiperazine-1,4-diium dihydrogen diphosphate Special details

**Table:**

Geometry. All esds (except the esd in the dihedral angle between two l.s. planes) are estimated using the full covariance matrix. The cell esds are taken into account individually in the estimation of esds in distances, angles and torsion angles; correlations between esds in cell parameters are only used when they are defined by crystal symmetry. An approximate (isotropic) treatment of cell esds is used for estimating esds involving l.s. planes.

### trans-2,5-Dimethylpiperazine-1,4-diium dihydrogen diphosphate Fractional atomic coordinates and isotropic or equivalent isotropic displacement parameters (Å^2^)

**Table:**

	*x*	*y*	*z*	*U*_iso_*/*U*_eq_	
P1	0.64197 (4)	0.21184 (5)	0.26767 (3)	0.02554 (12)	
O1	0.72741 (18)	0.0974 (2)	0.20950 (10)	0.0587 (5)	
O2	0.67285 (11)	0.20328 (14)	0.37398 (7)	0.0287 (2)	
O3	0.64629 (15)	0.37300 (17)	0.22218 (10)	0.0449 (3)	
O4	0.5000	0.1402 (2)	0.2500	0.0500 (6)	
H1	0.757 (3)	0.012 (4)	0.240 (2)	0.084 (10)*	
N1	0.61378 (12)	0.40835 (16)	0.52268 (9)	0.0238 (3)	
C1	0.62451 (15)	0.57743 (19)	0.49245 (11)	0.0248 (3)	
C2	0.51107 (15)	0.62671 (18)	0.42671 (10)	0.0239 (3)	
C3	0.5174 (2)	0.8018 (2)	0.40212 (19)	0.0443 (5)	
H1A	0.683 (2)	0.385 (3)	0.5625 (16)	0.042 (6)*	
H1B	0.622 (2)	0.345 (3)	0.4707 (16)	0.039 (6)*	
H1C	0.6259 (18)	0.642 (2)	0.5518 (14)	0.028 (5)*	
H1D	0.7042 (19)	0.588 (2)	0.4565 (14)	0.032 (5)*	
H2	0.510 (2)	0.559 (3)	0.3668 (15)	0.039 (5)*	
H3A	0.516 (3)	0.862 (4)	0.460 (2)	0.084 (10)*	
H3B	0.442 (3)	0.833 (3)	0.359 (2)	0.064 (8)*	
H3C	0.603 (3)	0.817 (3)	0.3694 (19)	0.061 (7)*	

### trans-2,5-Dimethylpiperazine-1,4-diium dihydrogen diphosphate Atomic displacement parameters (Å^2^)

**Table:**

	*U*^11^	*U*^22^	*U*^33^	*U*^12^	*U*^13^	*U*^23^
P1	0.0296 (2)	0.0287 (2)	0.01809 (17)	0.00277 (15)	−0.00450 (13)	0.00137 (14)
O1	0.0792 (12)	0.0686 (11)	0.0291 (7)	0.0334 (9)	0.0148 (7)	−0.0004 (7)
O2	0.0323 (6)	0.0344 (6)	0.0189 (5)	0.0073 (5)	−0.0055 (4)	−0.0012 (4)
O3	0.0556 (9)	0.0387 (7)	0.0400 (7)	−0.0063 (6)	−0.0100 (6)	0.0169 (6)
O4	0.0415 (11)	0.0244 (9)	0.0823 (15)	0.000	−0.0347 (10)	0.000
N1	0.0225 (6)	0.0279 (6)	0.0210 (5)	0.0053 (5)	−0.0025 (4)	0.0010 (5)
C1	0.0233 (6)	0.0276 (7)	0.0233 (6)	−0.0022 (5)	−0.0009 (5)	0.0008 (5)
C2	0.0252 (7)	0.0260 (7)	0.0206 (6)	0.0019 (5)	−0.0006 (5)	0.0031 (5)
C3	0.0479 (11)	0.0303 (9)	0.0548 (12)	0.0016 (8)	0.0029 (10)	0.0144 (9)

### trans-2,5-Dimethylpiperazine-1,4-diium dihydrogen diphosphate Geometric parameters (Å, º)

**Table:**

P1—O2	1.4916 (11)	N1—C1	1.484 (2)
P1—O3	1.4924 (14)	N1—C2^ii^	1.5020 (19)
P1—O1	1.5389 (15)	C1—C2	1.514 (2)
P1—O4	1.5884 (8)	C2—N1^ii^	1.5020 (19)
O4—P1^i^	1.5884 (8)	C2—C3	1.510 (2)
			
O2—P1—O3	116.60 (8)	P1—O4—P1^i^	135.50 (12)
O2—P1—O1	111.76 (8)	C1—N1—C2^ii^	112.80 (12)
O3—P1—O1	108.96 (9)	N1—C1—C2	111.58 (12)
O2—P1—O4	107.67 (5)	N1^ii^—C2—C3	109.69 (14)
O3—P1—O4	108.40 (8)	N1^ii^—C2—C1	108.68 (11)
O1—P1—O4	102.44 (9)	C3—C2—C1	111.29 (15)

Symmetry codes: (i) −*x*+1, *y*, −*z*+1/2; (ii) −*x*+1, −*y*+1, −*z*+1.

### trans-2,5-Dimethylpiperazine-1,4-diium dihydrogen diphosphate Hydrogen-bond geometry (Å, º)

**Table:**

*D*—H···*A*	*D*—H	H···*A*	*D*···*A*	*D*—H···*A*
O1—H1···O3^iii^	0.88 (3)	1.61 (3)	2.460 (2)	161 (3)
N1—H1*A*···O2^iv^	0.90 (2)	1.86 (2)	2.7454 (17)	167 (2)
N1—H1*B*···O2	0.90 (2)	1.87 (2)	2.7543 (17)	168 (2)
C1—H1*C*···O3^v^	0.982 (19)	2.353 (19)	3.193 (2)	143.0 (15)
C1—H1*D*···O1^vi^	0.970 (19)	2.41 (2)	3.205 (2)	139.1 (15)
C2—H2···O3^i^	1.00 (2)	2.53 (2)	3.344 (2)	138.3 (15)

Symmetry codes: (i) −*x*+1, *y*, −*z*+1/2; (iii) −*x*+3/2, *y*−1/2, −*z*+1/2; (iv) −*x*+3/2, −*y*+1/2, −*z*+1; (v) *x*, −*y*+1, *z*+1/2; (vi) −*x*+3/2, *y*+1/2, −*z*+1/2.
